# Okra WRKY Transcription Factor AeWRKY32 and AeWRKY70 Are Involved in Salt Stress Response

**DOI:** 10.3390/ijms252312820

**Published:** 2024-11-28

**Authors:** Jiaojun He, Shutong Chen, Ru Chen, Xinyu Li, Jiahua Wu, Yueping Zheng, Feifei Li, Yihua Zhan

**Affiliations:** College of Advanced Agricultural Sciences, Zhejiang A & F University, Hangzhou 311300, China; hejiaojun@stu.zafu.edu.cn (J.H.); 19858103506@163.com (S.C.); chenru_0@163.com (R.C.); 13575314707@163.com (X.L.); 19550176782@163.com (J.W.); ZhengYP@zafu.edu.cn (Y.Z.); lifei-fei@163.com (F.L.)

**Keywords:** *Abelmoschus esculentus*, salt stress, WRKY transcription factor, reactive oxygen species, antioxidant enzymes

## Abstract

Soil salinization is one of the abiotic stresses that inhibit plant growth and development, which seriously restricts global crop production. WRKY transcription factors play an important role in regulating plant responses to stress such as salt stress. In our previous study, two WRKY family genes from okra, *AeWRKY32* and *AeWRKY70*, were significantly up-regulated and down-regulated, respectively, in response to salt stress. In this study, subcellular localization showed that they were localized to the nucleus. The down-regulation of *AeWRKY32* and *AeWRKY70* via whole plant virus-induced gene silencing (VIGS) increased and decreased plant sensitivity to salt stress, respectively. Ectopic expression of *AeWRKY32* and *AeWRKY70* led to promoted and reduced salt tolerance in transgenic *Arabidopsis*, respectively. There was no significant difference between transgenic plants and wild type (WT) without salt treatment. Salt stress significantly inhibited plant growth. The decrease of chlorophyll content and the increase of anthocyanin content in *AeWRKY32*-overexpressed transgenic plants were lower than those in the WT, while *AeWRKY70*-overexpressed plants had the opposite effect. Under salt stress, the *AeWRKY70*-overexpressed plants had the highest malondialdehyde (MDA) content, followed by the WT, and the lowest in *AeWRKY32*-overexpressed plants. The hydrogen peroxide (H_2_O_2_) content and superoxide anion (O_2_^•−^) generation rate were only slightly increased. Moreover, salt stress significantly increased plant proline content and antioxidant enzyme activities, which was highest in *AeWRKY70*-overexpressed plants except superoxide dismutase (SOD). Taken together, these results suggest that AeWRKY32 and AeWRKY70 play positive and negative roles in plant in response to salt stress, respectively.

## 1. Introduction

With the increase in irrational human activities, changes in the natural environment and other factors lead to the accumulation of salt minerals and soluble salts in the soil, making soil salinization increasingly serious. Salt stress has become one of the most important abiotic stresses in modern agriculture, which has a negative impact on the morphogenesis, growth and development of plants, and restricts the sustainable development of agricultural production [1]. Salt accumulation can cause crops to be subjected to osmotic stress and ion stress, affect crop root water absorption and stomatal closure, inhibit crop growth, and even cause ion toxicity [2]. Saline soil also hinders seed water absorption, inhibits seed germination, affects the sugar signaling pathway and content in crops, as well as photosynthesis, and restricts the growth and development of crops [3,4]. The contents of malondialdehyde (MDA), superoxide dismutase (SOD), and peroxidase (POD) in the leaves of plants under salt stress gradually increased with the extension of stress time [5]. At this time, soluble protein, soluble sugar and vitamin C contents decreased. When plants are subjected to salt stress, they will cause the massive generation of reactive oxygen species (ROS), thus causing oxidative damage to plants. On the other hand, stress can promote the production of plant secondary metabolites. Anthocyanin is a class of flavonoids that is widely distributed in plants. Plants produce anthocyanin through a series of regulation and expression of anthocyanin synthesis genes, thereby eliminating ROS and reducing tissue damage [6].

Plants have evolved various mechanisms to cope with salt stress, such as regulating the expression of downstream genes through transcription factors in response to salt stress. Studies have found that WRKY and other transcription factors are crucial for plant salt stress response [7]. The WRKY transcription factor (TF) family is one of the largest transcription factor families in plants and plays an important role in vegetative growth, reproductive growth, development, and stress response of plants [8]. The name of WRKY TF derives from its unique highly conserved WRKY domain consisting of 60 amino acids, which is the DNA-binding region of the WRKY protein, including a highly conserved WRKYGQK amino acid sequence at the N terminus and a zinc finger motif at the C terminus [9]. The C-terminal zinc finger motif is a finger-like DNA binding domain formed by binding a zinc atom with conserved cysteine (Cys) and histidine (His) [10]. WRKY domain can bind to the promoter region of downstream target genes and regulate the expression of downstream genes.

Ishiguro and Nakamura [11] cloned *SPF1*, the first WRKY family gene, from the sweet potato (*Ipomoea batatas*). Subsequently, WRKY family members were identified in plants such as *Arabidopsis* [12], wheat [13], legumes [14] and eggplant [15]. Studies have shown that WRKY TF genes play an important role in the salt stress response of a variety of plants. Overexpression of upland cotton *GhWRKY34* in *Arabidopsis* enhanced the salt tolerance of transgenic plants [16]. In *Arabidopsis*, AtWRKY57 is involved in the response of ABA and drought stress [17], and AtWRKY25 and AtWRKY33 are involved in the regulation of salt stress [18]. Rice OsWRKY54 can bind to *OsHKT1;5* promoters and play a positive regulatory role in salt tolerance [19]. FcWRKY70 improves the salt tolerance of plants by promoting the expression of arginine decarboxylase [20]. As a positive regulator, *Ipomoea batatas* IbWRKY47 regulates the expression of stress-related genes to improve resistance to salt stress [21]. In *Sorghum bicolor*, SbWRKY50 is involved in salt stress tolerance through binding to the SOS1 and HKT1 promoter to maintain ion homeostasis [22]. Additionally, some WRKYs play a negative regulatory role in salt stress resistance. Transgenic *Arabidopsis* overexpressing chrysanthemum *CmWRKY17* is more sensitive to salt stress [23]. The expression of GhWRKY68 decreased the salt tolerance of cotton [24].

As a medicinal and edible plant, *Abelmoschus esculentus* L. is rich in potassium, calcium, iron, zinc, manganese, and other nutritional elements [25] and has a large number of active substances that can effectively prevent and treat cardiovascular diseases, digestive diseases, etc., and has attracted people’s attention [26]. We focused on the response of okra to salt stress and found that NAC83 plays a regulatory role in plant growth and development and salt stress [27]. In addition, our previous study has found that *AeWRKY32* and *AeWRKY70*, two WRKY TF genes from okra, were significantly up-regulated and down-regulated under salt stress, respectively [28]. However, there are no reports on the effects of WRKY members in response to salt stress in okra. In this study, the coding sequences of *AeWRKY32* and *AeWRKY70* genes were cloned from the obtained full-length transcript sequences of okra, and the subcellular localization and their responses to salt stress were analyzed. By observing the phenotype and response analysis of *AeWRKY32* and *AeWRKY70*-knocked-down okra and transgenic *Arabidopsis* to salt stress, we explored their regulatory effects on plant growth and development and salt stress response. These studies will provide important gene resources for molecular breeding of plant stress resistance.

## 2. Results

### 2.1. Expression Analysis of AeWRKY32 and AeWRKY70 in Okra Seedlings Exposed to Salt Stress

**Figure 1:**
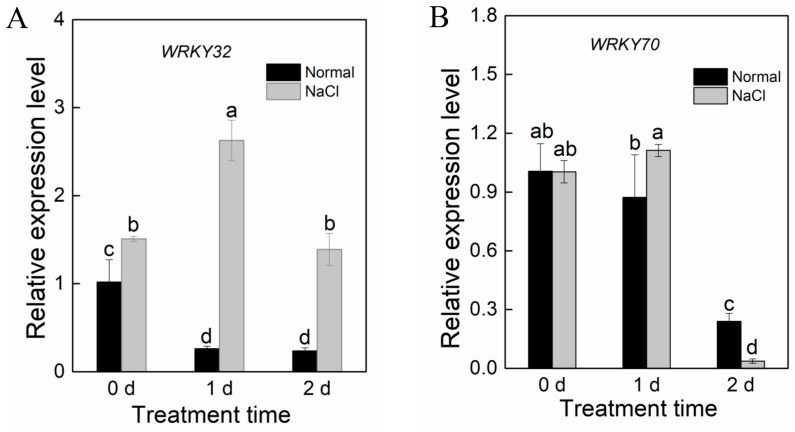
Expression of *AeWRKY32* (**A**) and *AeWRKY70* (**B**) in two-week okra seedlings exposed to 300 mM NaCl treatment by RT-qPCR. Data are shown as mean ± SD (*n* = 3). Different letters indicate significant differences (*p* < 0.05) using variance (ANOVA) and Duncan’s multiple range test.

Two-week okra seedlings were treated with salt treatment, and the second true leaf was sampled at three time points (0, 1, and 2 days after treatment) to determine the relative expression of *AeWRKY32* and *AeWRKY70* genes by RT-qPCR (Figure 1). The results showed that the expression of *AeWRKY32* was significantly up-regulated after salt treatment compared to the control, with the highest expression on day 1, which was about 10-fold higher than that of the control (Figure 1A). The expression of *AeWRKY70* was slightly up-regulated after 1 day of treatment and significantly down-regulated at 2 days (Figure 1B). These results suggest that AeWRKY32 and AeWRKY70 may play positive and negative regulatory roles, respectively, in plants in response to salt stress.

### 2.2. Molecular Cloning, Subcellular Localization of AeWRKY32 and AeWRKY70

Using the full-length transcript sequences of okra, the coding sequences of *AeWRKY32* and *AeWRKY70* genes were cloned. *AeWRKY32* and *AeWRKY70* encode 187 and 279 amino acids, respectively. Phylogenetic tree and multiple sequence alignment showed that they were distantly related to *Arabidopsis* (Figures S1 and S2). AeWRKY32 and *Arabidopsis* group I WRKY TF AtWRKY32 shared 16.9% amino acid sequence identity (Figure S2A). AeWRKY70 is homologous to *Arabidopsis* group III WRKY TFs AtWRKY54 and AtWRKY70 and shares approximately 7% amino acid sequence similarity (Figure S2B).

**Figure 2:**
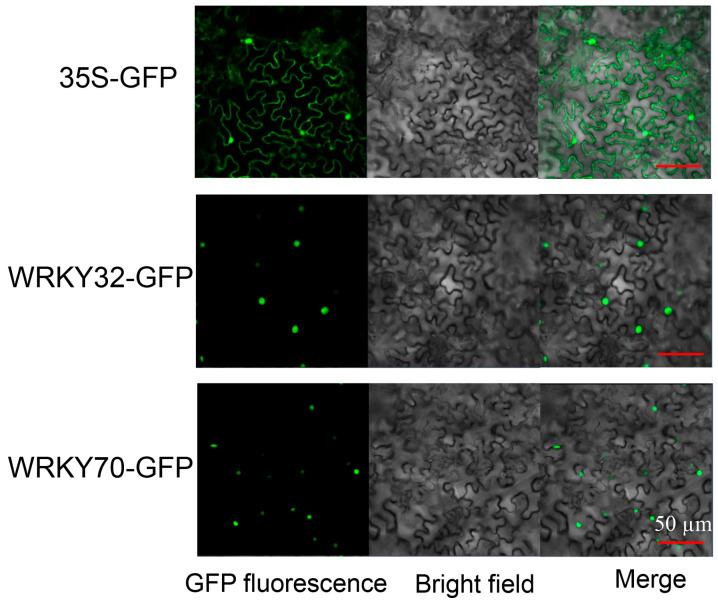
Subcellular localization of AeWRKY32 and AeWRKY70 in *N. benthamiana*.

The constructed *AeWRKY32*-GFP and *AeWRKY70*-GFP fusion proteins were transiently expressed in tobacco epidermal cells. The results showed that the tobacco epidermal cells with the empty vector had green fluorescence in both the nucleus and cytoplasm, while the epidermal cells with the fusion protein only had green fluorescence in the nucleus (Figure 2). These results suggest that AeWRKY32 and AeWRKY70 proteins were located in the nucleus, supporting the hypothesis that they were transcription factors.

### 2.3. Performance of AeWRKY32- and AeWRKY70-Knocked-down Plants Exposed to Salt Stress

**Figure 3:**
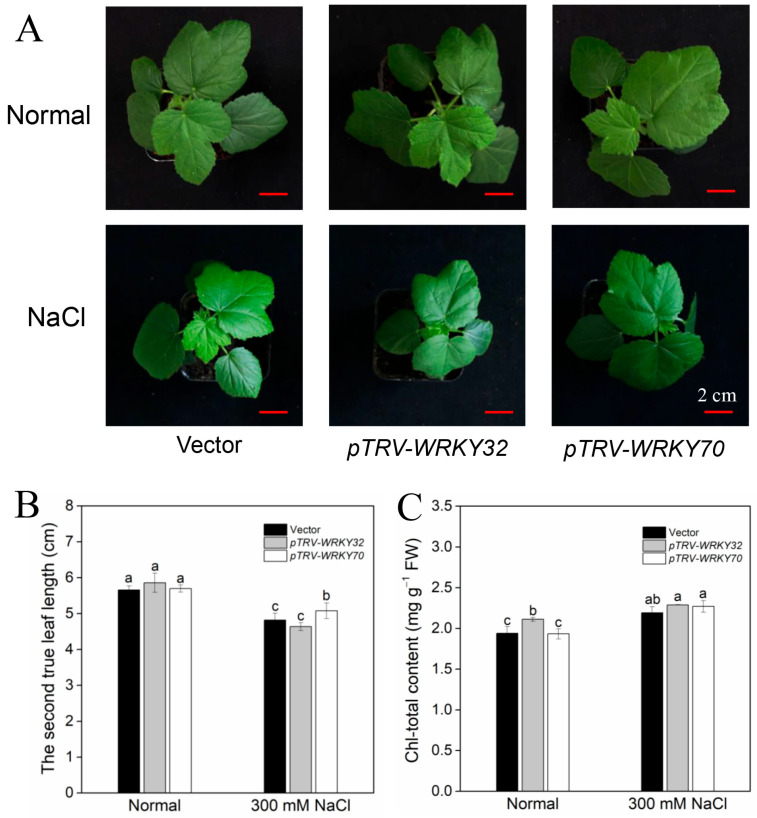
Performance of *AeWRKY32*- and *AeWRKY70*-knocked-down okra plants exposed to 300 mM NaCl treatment. (**A**) Phenotypes. (**B**) The second true leaf length. (**C**) Total chlorophyll content. Different letters indicate significant differences (*p* < 0.05) using variance (ANOVA) and Duncan’s multiple range test.

In order to observe the function of AeWRKY32 and AeWRKY70 in plant responses to salt stress, they were silenced in okra using virus-induced gene silencing (VIGS). As shown in Figure S3, compared with the empty vector, the mRNA levels of *AeWRKY32* and *AeWRKY70* in silenced plants declined, accounting for about 66 and 50% of the control, respectively, indicating that the genes were only knocked down. Then, the resulting *AeWRKY32*- and *AeWRKY70*-knocked-down okra seedlings were treated with 300 mM NaCl for 7 days. Without salt treatment, *AeWRKY32*-knocked-down okra seedlings were slightly larger than those of the control (Figure 3A). While the *AeWRKY70*-knocked-down seedlings were not significantly different from those of the control, and there was no significant change in the second true leaf length (Figure 3B). Under salt treatment, the growth of seedlings was significantly inhibited. The size of *AeWRKY32*-knocked-down okra seedlings decreased more than that of seedlings knocked down with empty vector only (Figure 3A), indicating that *AeWRKY32*-knocked-down plants were more sensitive to salt stress. On the contrary, the *AeWRKY70*-knocked-down okra seedlings showed a significant increase in size, with an increase in the length of the second true leaf, indicating that *AeWRKY70*-knocked-down plants have more tolerance to salt stress.

Chlorophyll is one of the key photosynthetic pigments in plants and other photosynthetic organisms, which is closely related to plant stress resistance. Herein, the chlorophyll content of *AeWRKY32*- and *AeWRKY70*-knocked-down okra seedlings and the control were measured. Under normal conditions, the total chlorophyll content in the *AeWRKY32*-knocked-down plants was higher than that in the control (Figure 3C), while the total chlorophyll of *AeWRKY70*-knocked-down plants was not significantly different from that of the control (Figure 3C). Under salt treatment, the total chlorophyll content in the control and gene-knocked-down seedlings increased significantly, the highest in the *AeWRKY70*-knocked-down plants. The results taken together indicated that AeWRKY32 and AeWRKY70 may play a positive and negative regulatory role in salt stress, respectively.

### 2.4. Performance of AeWRKY32- and AeWRKY70-Overexpressed Plants Exposed to Salt Stress

**Figure 4:**
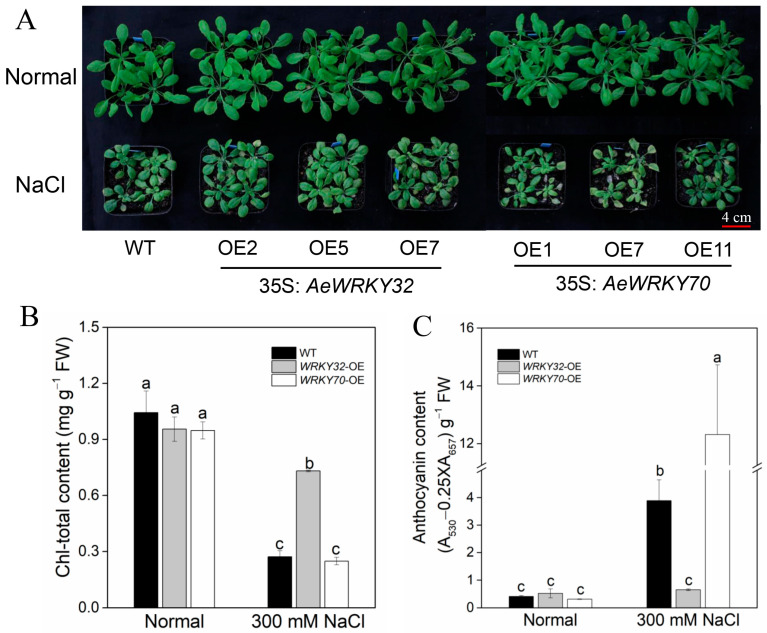
Performance of *AeWRKY32*- and *AeWRKY70*-overexpressed plants exposed to 300 mM NaCl treatment. (**A**) Phenotypes. (**B**) Total chlorophyll content. (**C**) Anthocyanin content. Different letters indicate significant differences (*p* < 0.05) using variance (ANOVA) and Duncan’s multiple range test.

To further explore the function of AeWRKY32 and AeWRKY70 in plant response to salt stress, they were overexpressed in *Arabidopsis thaliana*. As shown in Figure S4, compared with the WT, the mRNA levels of *AeWRKY32* and *AeWRKY70* in transgenic plants increased markedly, indicating that the genes were overexpressed. The homozygous transgenic lines with higher expression were selected for salt stress response analysis. The results showed that there was no significant difference in growth and development between the untreated transgenic seedlings and WT (Figure 4A), as well as in chlorophyll content (Figure 4B) and anthocyanin content (Figure 4C). Salt stress significantly inhibited the growth of all seedlings, with *AeWRKY32*-overexpressed plants growing better compared to WT, while *AeWRKY70*-overexpressed plants grew weaker, and old leaves turned yellow (Figure 4A). Under salt stress, chlorophyll content was significantly decreased, and WT and *AeWRKY70*-overexpressed plants decreased more than that of the *AeWRKY32*-overexpressed plants, accounting for 60 to 70% (Figure 4B). The anthocyanin content in WT and *AeWRKY70*-overexpressed plants increased significantly, and the increase in *AeWRKY70*-overexpressed plants was significantly greater than that in WT plants (Figure 4C). However, the anthocyanin content in *AeWRKY32*-overexpressed plants was only slightly increased, and there was no significant difference (Figure 4C). These results further validate their involvement in plant salt stress response.

### 2.5. Oxidative Stress of AeWRKY32 and AeWRKY70-Overexpressed Plants to Salt Stress

**Figure 5:**
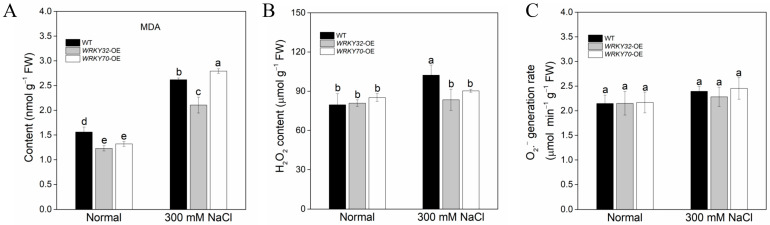
Malondialdehyde (MDA) content (**A**), hydrogen peroxide (H_2_O_2_) content (**B**) and super oxide (O_2_^•−^) generation rate (**C**) of *AeWRKY32*- and *AeWRKY70*-overexpressed plants exposed to 300 mM NaCl treatment. Different letters indicate significant differences (*p* < 0.05) using variance (ANOVA) and Duncan’s multiple range test.

MDA is a lipid peroxidation product, which is usually used as one of the indicators to measure the degree of oxidative damage to cells. The content of MDA in untreated plants was lower, and the content of WT was slightly higher than that in transgenic plants. Under salt treatment, MDA content increased significantly, with the highest in *AeWRKY70*-overexpressed plants, followed by WT plants, and the lowest in *AeWRKY32*-overexpressed plants (Figure 5A). Additionally, salt stress resulted in excessive ROS production (Figure 5B,C). There was no significant difference in hydrogen peroxide (H_2_O_2_) content in untreated plants, which increased significantly only in WT after salt treatment (Figure 5B). However, the production rate of superoxide anion (O_2_^•−^) was only slightly increased under salt treatment and there was no significant difference (Figure 5C).

### 2.6. Response of Proline and Antioxidant System of AeWRKY32 and AeWRKY70-Overexpressed Plants to Salt Stress

**Figure 6:**
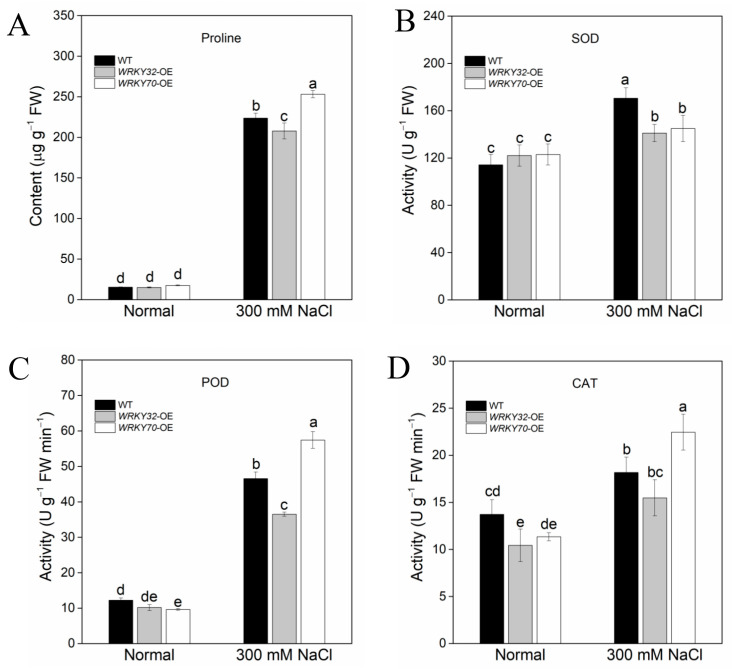
Proline content (**A**) and activities of superoxide dismutase (SOD) (**B**), peroxidase (POD) (**C**) and catalase (CAT) (**D**) of *AeWRKY32-* and *AeWRKY70*-overexpressed plants exposed to 300 mM NaCl treatment. Different letters indicate significant differences (*p* < 0.05) using variance (ANOVA) and Duncan’s multiple range test.

Proline content and antioxidant enzyme activities of transgenic plants exposed to salt stress were also measured. As shown in Figure 6A, there was no significant difference in proline content in untreated plants. Under salt stress, proline content increased significantly, and the highest proline content was observed in *AeWRKY70*-overexpressed plants, followed by WT plants, and the lowest in *AeWRKY32*-overexpressed plants (Figure 6A).

The contents of antioxidant enzymes SOD, POD, and catalase (CAT) were lower in untreated plants. Under salt stress, antioxidant enzyme activities were significantly increased, and POD and CAT contents were the highest in *AeWRKY70*-overexpressed plants. The mean values of SOD in WT, *AeWRKY32*-overexpressed and *AeWRKY70*-overexpressed plants were increased by 49.4, 15.6, and 17.8%, respectively (Figure 6B). The mean values of POD in WT, *AeWRKY32*-overexpressed and *AeWRKY70*-overexpressed plants were increased by 280.0, 258.9, and 497.55%, respectively (Figure 6C). The mean values of CAT in WT, *AeWRKY32*- and *AeWRKY70*-overexpressed plants were increased by 32.4, 48.3, and 98.0%, respectively (Figure 6D).

## 3. Discussion

WRKY TF is one of the largest TF families in plants and is involved in various physiological processes, mainly in plant stress responses. Its members have been reported to be involved in plant responses to high salt and drought stress [29]. In *Arabidopsis*, WRKY25 and WRKY33 improve the resistance of transgenic plants to high salt through a SOS-independent signaling pathway [18]. Transgenic rice with *OsWRKY45* overexpression can improve the resistance to high salt, drought, and disease [30,31]. In this study, we found that the expression of okra *AeWRKY32* was up-regulated, and *AeWRKY70* was down-regulated under salt stress (Figure 1), suggesting that they may be involved in the regulation of salt stress response. Therefore, we knocked down the two TFs in okra by VIGS technology and treated the resulting *AeWRKY32*- and *AeWRKY70*-knocked-down seedlings with salt treatment. It was found that *AeWRKY32*-knocked-down seedlings were smaller than the control after salt treatment, while *AeWRKY70*-knocked-down seedlings had increased leaf length and chlorophyll content (Figure 3). Overall, *AeWRKY32*-knocked-down okra seedlings were more sensitive to salt stress, while *AeWRKY70*-knocked-down seedlings were significantly resistant to salt stress, suggesting that WRKY32 and WRKY70 may play positive and negative roles in salt stress, respectively.

*Arabidopsis* AtWRKY32, which is homologous with AeWRKY32 (Figure S1), also known as CSU5, is involved in light photomorphogenesis [32]. The involvement of WRKY32 in abiotic stress has been reported in other species. For instance, the overexpression of *IgWRKY50* and *IgWRKY32* from *Iris germanica* in *Arabidopsis* enhanced its resistance to drought stress by the ABA-mediated signal pathway [33]. As a positive regulator, *Verbena bonariensis* VbWRKY32 enhanced the resistance of plants to cold stress [34]. AeWRKY70 is homologous with *Arabidopsis* AtWRKY54 and AtWRKY70. AtWRKY54 and AtWRKY70 are closely related and have been proven to be the key components of plant response to biotic stress and involved in leaf senescence [35,36,37,38,39]. In recent years, their role in plant response to abiotic stress resistance has also been demonstrated. The *wrky54wrky70* double mutant enhanced the resistance of *Arabidopsis* to osmotic stress by regulating stomatal aperture [40]. Together with AtWRKY46, they also positively regulated BR-mediated growth and development and negatively regulated drought responses [41]. These findings are consistent with our results that AeWRKY70 is negatively involved in salt stress responses (Figure 3 and Figure 4).

Although we initially analyzed the roles of WRKY32 and WRKY70 in salt stress using VIGS technology, due to the complexity of the okra genome, only part of the *WRKY32* or *WRKY70* gene was silenced (Figure S3), so we further verified their functions in *Arabidopsis*. After salt treatment, transgenic plants overexpressing *AeWRKY32* and *AeWRKY70* enhanced the resistance and sensitivity to salt stress, respectively (Figure 4A). The chlorophyll content of WT decreased significantly under salt treatment, which was similar to that of *AeWRKY70* overexpression plants. However, the decrease in chlorophyll in *AeWRKY32* overexpression plants was significantly lower than that in WT plants (Figure 4B). Anthocyanin is a class of flavonoids that maintain the function of antioxidant enzymes during antioxidant processes. In addition, anthocyanin also has the ability to eliminate free radicals, thereby reducing the level of ROS by interacting with molecules in other signaling pathways. Anthocyanin content was significantly increased in plants under salt stress [42,43], and the highest content was observed in *AeWRKY70* overexpression plants (Figure 4C), which may be due to it being more sensitive to salt stress and producing more anthocyanins.

Under salt stress, plant cells will suffer from water shortage and ion imbalance, leading to osmotic stress [44]. MDA is usually used as the indicator to evaluate the degree of oxidative damage to cells. Under salt treatment, MDA content increased significantly, with the highest in *AeWRKY70*-overexpressed plants, followed by WT plants, and the lowest in *AeWRKY32*-overexpressed plants (Figure 5A), indicating overexpression of *AeWRKY32* conferred *Arabidopsis* resistance to salt stress, while *AeWRKY70*-overexpressed seedlings were more sensitive to salt stress. ROS, including O_2_^•−^, hydroxyl free radical (-OH) and H_2_O_2_ [45], are involved in the regulation of cell growth and development and may trigger oxidative stress in cells, thus affecting the physiological state and adaptability of plants [46]. When plants are subjected to abiotic stress, the probability of electron transfer to oxygen in the photosynthetic chain increases, leading to the massive production of ROS in mitochondria, chloroplasts, and peroxisomes [47,48]. Excessive ROS can negatively affect cellular structure and function. The H_2_O_2_ content and O_2_^•−^ generation rate increased in plants under salt stress, while *AeWRKY32*-overexpressed plants showed little change (Figure 5B,C).

During their long-term evolution, plants have gradually established a set of efficient mechanisms to remove excessive ROS, thereby maintaining the ROS homeostasis in the organism, which mainly includes two mechanisms, namely enzymatic reaction and non-enzymatic reaction [49]. Enzymatic reaction mainly involves a series of antioxidant enzymes, such as SOD, CAT, POD, etc. These enzymes remove peroxides and protect cells from oxidative damage by catalyzing the conversion of ROS. Under salt stress, the activities of SOD, CAT, and POD increased appreciably in WT and transgenic plants (Figure 6B–D). Additionally, in order to alleviate stress response, plants will synthesize important osmotic regulatory substances like soluble sugar and proline to maintain the osmotic balance of cells so as to maintain low cell turgor and normal operation of physiological activities in vivo to adapt to changes in the external environment [50]. Proline plays an important role as an osmotic regulator in plants. When plants face stress, such as salt stress or drought, proline synthesis is significantly increased [51,52,53], which is consistent with our results (Figure 6A). This accumulation not only helps to maintain the osmotic balance of cells and slow down water loss but also has significant antioxidant properties that help to mitigate the adverse effects of oxidative stress on plants.

## 4. Materials and Methods

### 4.1. Treatment of Okra Seedlings and RT-qPCR Analysis

In this study, okra seeds “Xianzhi” were selected, and the plant growth conditions were based on our previous study [28]. Two-week okra seedlings were selected for salt treatment and irrigated with 300 mM NaCl solution, while the control group was irrigated with water. The second true leaf was harvested at 0, 1, and 2 d after treatment. Total RNA was extracted to synthesize first-strand cDNA. The expression of *AeWRKY32* and *AeWRKY70* was analyzed by RT-qPCR. The primers used were shown in our previous study [28].

### 4.2. Subcellular Localization

The coding sequence (without terminator) of *AeWRKY32* and *AeWRKY70* genes were amplified from the cDNA of okra leaves and ligated into the pCAMBIA1300-sGFP vector to form 35Sp::*AeWRKY32*:GFP and 35Sp::*AeWRKY70*:GFP fusion proteins. The obtained plasmid and empty vector were transferred into *Agrobacterium* EHA105 to infect tobacco epidermal cells. After 2 days of tobacco cultivation, the distribution of green fluorescence was observed using a laser scanning confocal microscope (Zeiss LSM 710, Carl Zeiss, Oberkochen, Germany). The primers for *AeWRKY32* and *AeWRKY70* were 5′-TCTAGAGACGAACTCACTTTCATCAA-3′ and 5′-TCTAGAGCATGGCTTGATTTCGAATCC-3′, and 5′-GAGCTCATGGGAAGTGTGTCAGCTTG-3′ and 5′-TCTAGAGACGAACTCACTTTCATCAA-3′, respectively.

### 4.3. Acquisition and Treatment of Gene Silenced Plants by VIGS

About 300 bp of specific coding sequences of *AeWRKY32* and *AeWRKY70* genes were amplified and ligated into the *EcoR* I/*Kpn* I site of pTRV2 vector to form TRV-*WRKY32* and TRV-*WRKY70* vectors, which were transformed into *Agrobacterium* GV3101. The primers for *AeWRKY32* and *AeWRKY70* were 5′-GAATTCAGCTGTAGATAATACAAATG-3′ and 5′-GGTACCCATGGCTTGATTTCGAATCC-3′, and 5′-TCTAGAACAGACTTGGACAGTAGCTT-3′ and 5′-GAGCTCATGGCTGCAGTGTTAGATGG-3′, respectively. The constructed vector and pTRV1 were then inoculated into okra cotyledon as described by our previous study [27].

*AeWRKY32*- and *AeWRKY70*-silenced okra seedlings were irrigated with 300 mM NaCl solution or water for 7 days. Plant phenotypes were observed, leaf fresh weight and second true leaf length were recorded, and chlorophyll content of the second true leaf was determined [28].

### 4.4. Acquisition and Treatment of Transgenic Arabidopsis Plants

The overexpression vectors pCAMBIA1301-35Sp::*AeWRKY32* and pCAMBIA1301- 35Sp::*AeWRKY70* were constructed and infected to *Arabidopsis* ecotype Columbia (WT). WT and homozygous transgenic seedlings were grown in soil for 20 d, then watered with 300 mM NaCl solution and cultured for 7 d to observe the phenotype and determine the chlorophyll content and anthocyanin content [27]. The leaves were frozen in liquid nitrogen and stored at −80 °C for subsequent determination of physiological parameters.

### 4.5. Determination of MDA, H_2_O_2_ and O_2_^•−^ Content

MDA content was measured as described by Hodges et al. [54]. About 0.5 g of leaves were ground with 10% (*v*/*v*) trichloroacetic acid (TCA). After centrifugation at 4000 rpm for 10 min, 1 mL of supernatant was added into 4 mL of 20% TCA containing 0.65% (*v*/*v*) thiobarbituric acid (TBA). The mixture was heated at 95 °C for 25 min and immediately cooled. The supernatant was determined for absorbance at 440, 532, and 600 nm.

For the measurement of H_2_O_2_ [55], samples were ground with acetone precooled at 4 °C and centrifuged at 3000 rpm for 10 min. Next, 0.1 mL 5% titanium sulfate and 0.2 mL concentrated ammonia water were added into 1 mL supernatant. After precipitate formation, centrifuged at 3000 rpm for 10 min, and the supernatant was discarded. Additionally, 5 mL of sulfuric acid at a concentration of 2 M was added to the precipitate, and the absorbance at 415 nm was determined after dissolution.

The generation rate of O_2_^•−^ was determined as described by Li et al. [5].

### 4.6. Determination of Proline Content

First, 0.3 g of leaves were added with 5 mL of 3 g/L sulfosalicylic acid and heated at 95 °C for 10 min. Next, 2 mL of the filtrate, 2 mL of acetic acid, and 3 mL of acidic indanone were mixed and heated at 95 °C for 40 min. After cooling, 5 mL of toluene was added and shaken thoroughly. The upper layer of toluene solution was used to measure the absorbance at 520 nm.

### 4.7. Determination of Antioxidant Enzyme Activity

Approximately 0.5 g of leaves were ground with 5 mL of 0.05 M phosphate buffer (pH 7.8). After centrifugation at 10,000 rpm for 20 min at 4 °C, the obtained supernatant was the crude enzyme solution. Antioxidant enzyme (SOD, POD, and CAT) activity was determined according to the method of Hou et al. [56].

### 4.8. Statistical Analysis

Statistical analysis was performed with SPSS 19.0 (SPSS Inc., Chicago, IL, USA). Data are shown as mean ± SD (*n* = 3). Significant differences (*p* < 0.05) were determined using variance (ANOVA) and Duncan’s multiple range test.

## 5. Conclusions

In summary, *AeWRKY32* was up-regulated during salt stress, while *AeWRKY70* was down-regulated by RT-qPCR. Subcellular localization in the nucleus further supports that they are TFs. *AeWRKY32-* and *AeWRKY70*-knocked-down okra plants were more sensitive and resistant to salt stress. Overexpression of *WRKY32* in *Arabidopsis thalania* does appear to somewhat protect from salt stress, while *WRKY70* overexpression plants are smaller, with high anthocyanin content, marginally higher MDA content, slightly increased proline, and higher POD and CAT activity. These data suggest a role for AeWRKY32 and AeWRKY70 genes in regulating salt stress in okra. However, the effects of these two genes on salt stress were not so strong, probably due to the existence of other genes with high homology in okra and functional redundancy, which need to be further validated in subsequent studies.

## Data Availability

The original contributions presented in the study are included in the article/Supplementary Material; further inquiries can be directed to the corresponding author.

## Supplementary Materials

The following supporting information can be downloaded at: https://www.mdpi.com/article/10.3390/ijms252312820/s1.
