# Supplementary material for: Okra WRKY Transcription Factor AeWRKY32 and AeWRKY70 Are Involved in Salt Stress Response
Source: Int J Mol Sci. 2024 Nov 28;25(23):12820. doi: 10.3390/ijms252312820 (PMC11640966; doi:10.3390/ijms252312820)
Supplement: Supplementary file 1 [file ijms-25-12820-s001.zip › Figure S2.pdf]

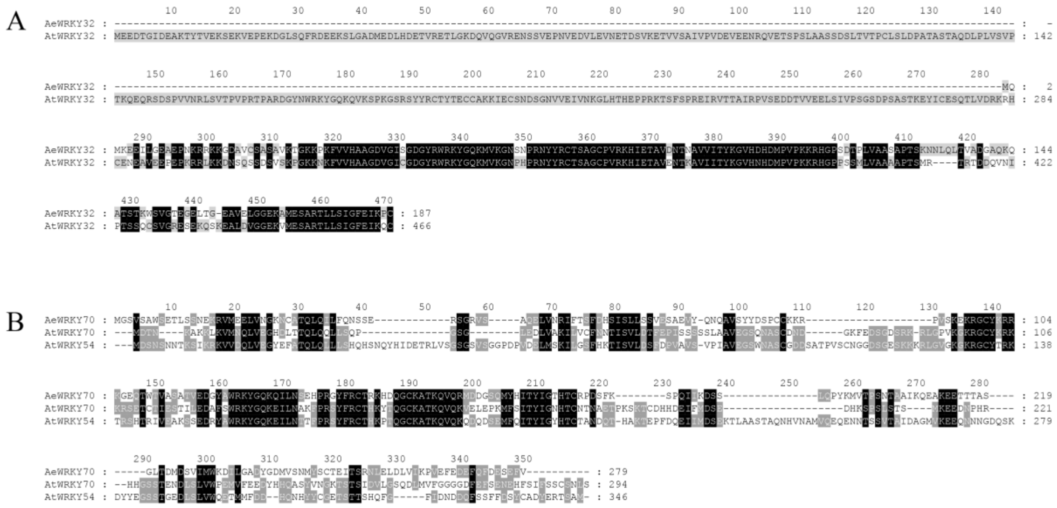

Figure S2. Multiple sequence alignment of AeWRKY32 and AeWRKY70 and its homologous *Arabidopsis thaliana* WRKY proteins. (**A**) AeWRKY32 and AtWRKY32. (**B**) AeWRKY70, AtWRKY70 and AtWRKY54.
