# Supplementary figures and images for: Okra WRKY Transcription Factor AeWRKY32 and AeWRKY70 Are Involved in Salt Stress Response
Source: Int J Mol Sci. 2024 Nov 28;25(23):12820. doi: 10.3390/ijms252312820 (PMC11640966; doi:10.3390/ijms252312820)

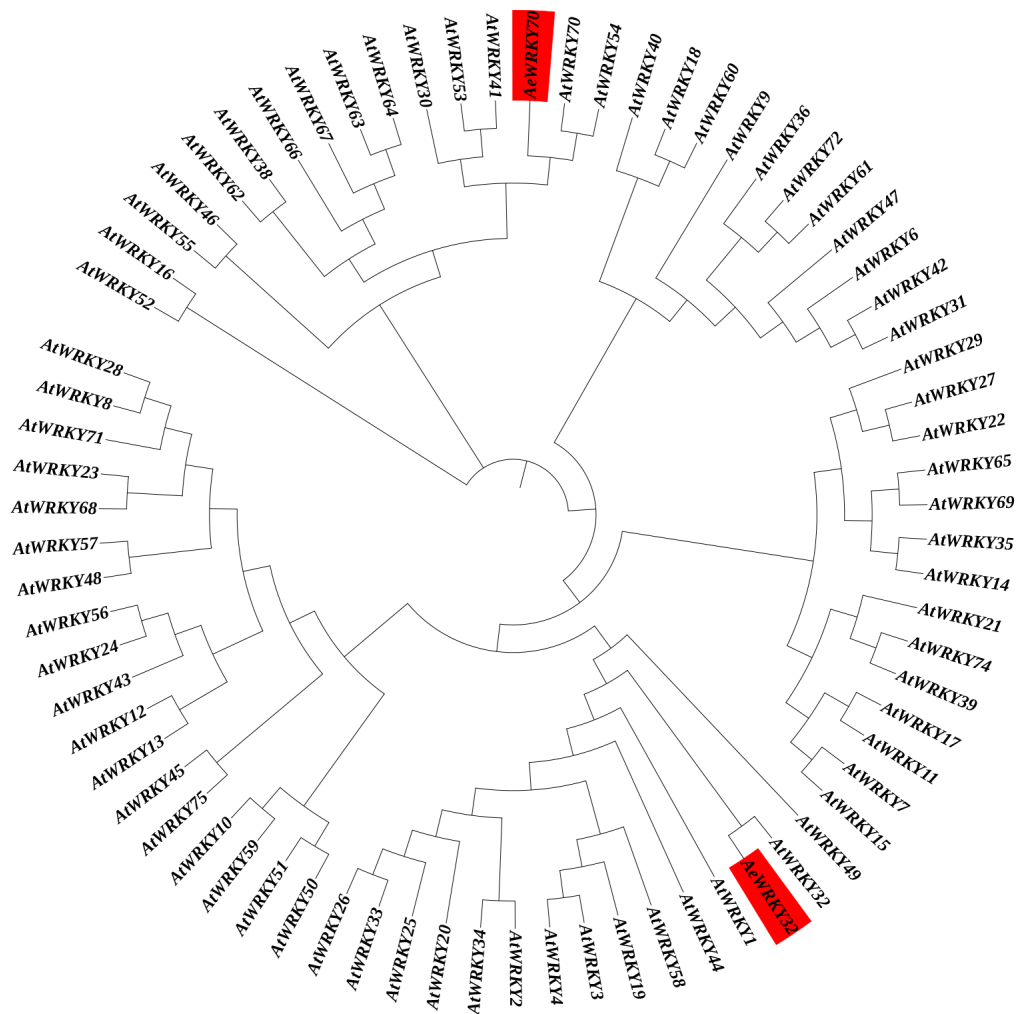

**Figure S1.** Phylogenetic analysis of *AeWRKY32* and *AeWRKY70* with *Arabidopsis thaliana* WRKY proteins.

Supplement: Supplementary file 1 [file ijms-25-12820-s001.zip › Figure S1.pdf]
